# Quick and effective improvement of leucine enriched dietary supplement on malnutrition in acute stroke patients receiving enteral tube feeding

**DOI:** 10.1186/s12873-020-00351-w

**Published:** 2020-07-20

**Authors:** Takahisa Mori, Kazuhiro Yoshioka

**Affiliations:** Department of Stroke Treatment, Shonan Kamakura General Hospital Stroke Center, Okamoto 1370-1, Kamakura City, Kanagawa 247-8533 Japan

**Keywords:** Severe stroke, Enteral tube feeding, Malnutrition, Transthyretin, Leucine, BCAA

## Abstract

**Background:**

Malnutrition often occurs in acute stroke patients receiving enteral tube feeding (ETF). Unless malnutrition is improved, their clinical outcome is poor. However, strategies to improve malnutrition in these patients have not been established. Branched-chain amino acids (BCAA) may enhance protein synthesis and attenuate inflammation. Our study aimed to investigate whether a leucine enriched BCAA dietary supplement (LEBDs) could quickly increase serum levels of albumin (Alb) or transthyretin (TTR) and decrease high-sensitivity C-reactive protein (CRP) in the development of severe malnutrition within a few days after stroke onset compared to standard BCAA dietary supplement (SBDs).

**Methods:**

We retrospectively included acute stroke patients who: 1) were admitted between August 2016 and July 2017; 2) underwent ETF for 7 days or longer after admission, and 3) underwent blood examination of Alb, TTR, and CRP on admission, the fifth day and the seventh day. We defined severe malnutrition as severe hypoproteinemia: decrease of TTR to less than 15 mg/dl on the 5th day. In LEBDs and SBDs groups, patients started to receive a dietary supplement containing leucine of 1.44 and 0. 72 g twice a day on the fifth day, respectively. We evaluated Alb (g/dl), TTR (mg/dl), and CRP (mg/dl) on admission, the fifth day, and the seventh day.

**Results:**

Twenty-nine patients met our inclusion criteria:15 in LEBDs and 14 in SBDs. In LEBDs and SBDs groups, the median Alb was 3.5 and 3.3 g/dl, TTR was 12.7 and 10.7 mg/dl, and CRP was 1.02 and 0.673 mg/dl on admission, respectively. In LEBDs, the median Alb and TTR decreased to 2.6 g/dl and 11.9 mg/dl, and CRP increased to 5.337 mg/dl on the fifth day. On the 7th day, TTR increased, and CRP decreased, although Alb did not improve. In SBDs, the median Alb and TTR decreased to 2.6 g/dl and 9.7 mg/dl, and CRP increased to 4.077 mg/dl on the fifth day. On the 7th day, Alb, TTR, and CRP did not improve.

**Conclusion:**

In acute stroke patients receiving leucine enriched BCAA dietary supplement, quick improvements in transthyretin and CRP were observed.

## Background

Malnutrition often occurs in severe acute stroke patients who are unable to take foods orally due to dysphagia or disturbed level of consciousness and who must receive enteral tube feeding (ETF) [1–3]. Malnutrition leads to the severity of the general condition and subsequently extended stay in hospital or in-hospital mortality [4–7]. Malnutrition may contribute to a weakened immune system and causes infection to occur [8]. Acute inflammation associated with infection exacerbates hypoproteinemia, and the clinical outcomes in patients with severe hypoproteinemia are generally poor. If malnutrition occurs in a patient, it should be treated and improved as soon as possible. Inflammation must be attenuated as soon as possible. However, it has not been established on how to improve hypoproteinemia rapidly [9]. Larger volumes of protein intake in the diet do not always lead to rapid improvement of hypoproteinemia and cessation of inflammation due to anabolic resistance in a severe clinical state [10, 11]. Leucine is one of the essential amino acids and one of the branched-chain amino acids (BCAA) [12], and previous studies have reported that leucine enriched BCAA supplement may attenuate inflammation, enhance protein synthesis [13–15] and may be useful in recovering muscle mass and strength and in promoting rehabilitation [13, 16–19]. Our study aimed to investigate whether leucine-enriched BCAA dietary supplement (LEBDs) could quickly increase serum levels of albumin (Alb) or transthyretin (TTR) and quickly decrease high-sensitivity C-reactive protein (CRP) in the development of severe malnutrition within a few days after stroke onset compared to standard BCAA dietary supplement (SBDs).

## Methods

For the retrospective observational study, we included acute stroke patients who: 1) were admitted between August 2016 and July 2017, 2) started ETF on the second day and continued to undergo ETF for 7 days or longer after admission, 3) underwent blood examination on admission, the fifth day and the seventh day, and 4) started to receive LEBDs or SBDs to improve severe malnutrition on the fifth day. We defined severe malnutrition as rapid progression of hypoproteinemia: decrease of TTR to less than 15 mg/dl, as TTR is a rapid turnover protein [20]. Patients started to receive LEBDs or SBDs twice a day on the fifth day added to baseline enteral nutrients.

### Exclusion criteria

We excluded from our analysis patients who: 1) died due to very severe stroke within 7 days after its onset, 2) could take foods orally on admission, or 3) did not undergo a thorough blood examination.

### Target calories

We calculated the target calories for patients according to the Harris-Benedict Equation (HBE) [21].

### Enteral nutrients

We started to administer enteral nutrients on the second day of admission. Enteral nutrients (EN) we used to be IMPACT (Nestle Japan Co. Ltd.), PEPTAMEN AF (Nestle Japan Co. Ltd.), MEIN (Meiji Co. Ltd., Japan) or RENALEN MP (Meiji Co. Ltd., Japan). IMPACT includes contents that consist of protein of 14 g (arginine-enhanced), containing leucine of 0.975 g, lipid of 7.0 g, containing middle-chain triglyceride (MCT) of 1.5 g, and carbohydrates of 33.5 g against 250 kcal per 250 ml of one pack. PEPTAMEN AF includes contents that consist of protein of 19 g, containing leucine of 2.03 g and whey peptide, lipid of 13.2 g, containing MCT of 6.7 g, and carbohydrates of 26.4 g against 300 kcal per 200 ml of one pack. MEIN consists of protein of 10 g, containing leucine of 0.96 g and whey peptide, lipid of 5.6 g, containing MCT of 1.2 g, and carbohydrates of 29.8 g, containing palatinose, against 200 kcal per 200 ml of one pack. Renalen MP consists of protein of 14 g, containing leucine of 1.32 g, lipid of 11.2 g, containing MCT of 2.3 g, and carbohydrates of 64.0 g, containing palatinose, against 400 kcal per 250 ml of one pack. Palatinose is a disaccharide that consists of one glucose and one fructose, and it has a low glycemic index. Enteral nutrients except for RENALEN MP were protein-rich nutrients. Leucine enriched BCAA dietary supplement (LEBDs) (Leucine Plus, Nestle, and Ajinomoto, Japan) consists of protein of 8 g, containing 1.44 g of leucine, and sugar of 18.5 g, against 200 kcal per 100 ml of one pack, and standard BCAA dietary supplement (SBDs) (Meibalance Mini, Meiji Co. Ltd., Japan) consists of protein of 7.5 g, containing leucine of 0.72 g and sugar of 29.3 g, against 200 kcal of one pack (Table 1). Protein volume in LEBDs was almost the same as in SBDs.

**Table 1:** Contents of leucine enriched and standard BCAA dietary supplements

Dietary supplement	Leucine enriched BCAA	Standard BCAA
Volume (1 pack)	100 ml	125 ml
Calories (1 pack)	200 kcal	200 kcal
Protein	8.0 g	7.5 g
BCAA	2.07 g	1.58
Leucine	**1.44 g**	0.72
Valine	0.36 g	0.48
Isoluecine	0.27 g	0.38
Lipid	10.3 g	5.6 g
MCT	3.3 g	no information
n-6 FA	2.1 g	no information
n-3 FA	0.3 g	no information
Carobohydrates	20.4 g	31.8 g
Sugar	18.5 g	29.3 g
Dietary fiber	1.9 g	2.5 g
Water	70 g	93.7 g
Na	110 mg	110 mg
K	127 mg	120 mg

*BCAA* Branched chain amino acid, *kcal* kcalorie, *MCT* Middle chain fatty acid, *FA* Fatty acid, *Na* Natrium, *K* Potassium

### Evaluation

We evaluated patient features, TTR, Alb, and high-sensitivity CRP on admission, the fifth day, the 7th day, because previous studies have reported CRP elevation and decrease in Alb or TTR in patients with severe inflammation or severe acute conditions [22–24]. Besides, we evaluated Glasgow Coma Scale (GCS) [25], in-hospital clinical outcome, hospitalization days, and serum Cre on admission, the fifth day, the 7th day, because deterioration of renal function was a possible adverse effect of protein-rich EN.

### Statistical analysis

Non-normally distributed continuous variables were expressed as the median and interquartile range (IQR). Differences between LEBDs and SBDs were compared using a Fisher’s exact test for categorical variables and a Wilcoxon rank-sum test for non-parametric data. For a comparison of paired variables, we used a Wilcoxon signed-rank test for non-parametric data. We performed a two-sided test for unpaired variables, a one-sided test for paired variables, and considered a probability of less than 0.05 statistically significant. We used the JMP (version 15.1) program to perform the statistical analysis.

## Results

During the study period, we treated 800 acute stroke patients in our institution. Among patients who underwent ETF on the second day, the TTR level on the fifth day was less than 15 mg/dl in 29 patients, and the 15 patients started to receive LEBDs and the 14 patients started to receive SBDs on the 5th day. There were no differences in patients’ characteristics between the two groups. Their median age was older than 80 years. In LEBDs and SBDs groups, the median GCS on admission was 9 and 9.5, respectively, and they suffered from impaired consciousness.

**Fig. 1:**
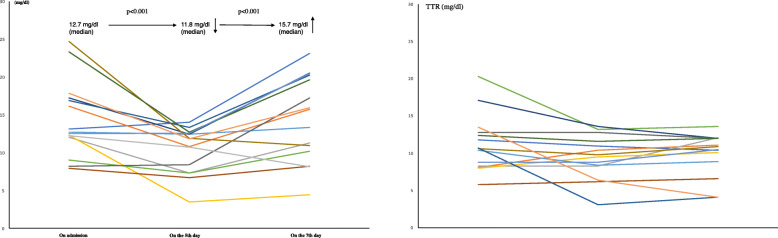
Serial changes of serum TTR on admission, on the fifth day, and the seventh day. One-sided Wilcoxon signed-rank test was performed. Left: Leucine enriched BCAA dietary supplement, Right: Standard BCAA dietary supplement, BCAA: branched-chain amino acid, TTR: transthyretin

**Fig. 2:**
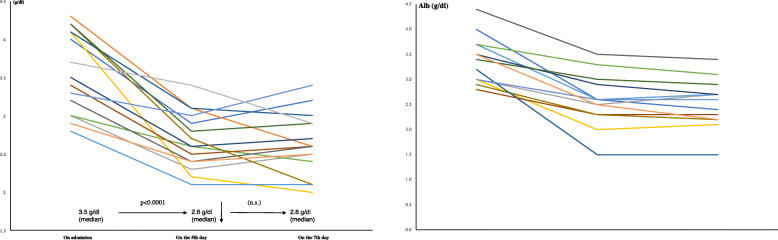
Serial changes of serum Alb on admission, on the fifth day and the seventh day. As shown, a one-sided Wilcoxon signed-rank test was performed. Left: Leucine enriched BCAA dietary supplement, Right: Standard BCAA dietary supplement, BCAA: branched-chain amino acid, Alb: albumin

**Fig. 3:**
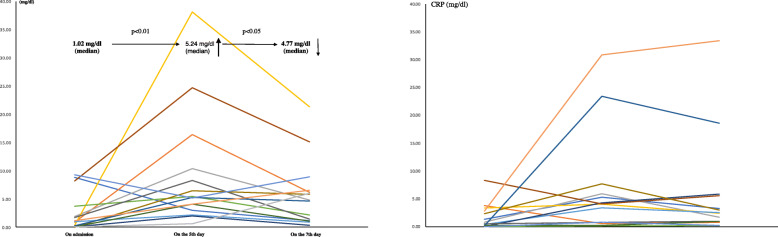
Serial changes of serum CRP on admission, on the fifth day and the seventh day. One-sided Wilcoxon signed-rank test was performed. Left: Leucine enriched BCAA dietary supplement, Right: Standard BCAA dietary supplement, BCAA: branched-chain amino acid, CRP: high-sensitivity C-reactive protein

In LEBDs and SBDs groups, the median Alb was 3.5 and 3.3 g/dl (ns), TTR was 12.7 and 10.7 mg/dl (ns), and CRP was 1.02 and 0.673 mg/dl (ns) on admission, respectively (Table 2). In LEBDs group, patients received baseline protein intake of 1.2 (g/kg/day) by enteral nutrition (Table 3). However, the median Alb decreased to 2.6 g/dl (*p* <  0.0001) and the median TTR decreased to 11.9 mg/dl (*p* <  0.001), and CRP increased to 5.337 mg/dl (*p* <  0.05) on the fifth day. We added the LEBDs (2.88 g of leucine/400 kcal/200 ml/day) to baseline EN. On the 7th day, TTR increased (*p* <  0.001) and CRP decreased (*p* <  0.05) although Alb did not improve (Figs. 1, 2 and 3; left) (Table 4). In SBDs group, patients received baseline protein intake of 1.4 (g/kg/day) by enteral nutrition (Table 3). However, the median Alb decreased to 2.6 g/dl (*p* <  0.0001) and the median TTR decreased to 9.7 mg/dl (*p* < 0.05), and the median CRP increased to 4.077 (*p* < 0.05) on the fifth day. We added the SBDs (1.44 g of leucine/400 kcal/250 ml/day) to baseline EN. On the 7th day, Alb, TTR and CRP did not improve (Figs. 1, 2 and 3; right) (Table 4). The serum TTR level on the 7th day was higher in LEBDs than in SBDs, although there were no differences in TTR on the 5th day between LEBDs and SBDs (Table 2).

**Table 2:** Patients’ characteristics in Leucine enriched and standard BCAA dietary supplements

Dietary supplement	Leucine enriched BCAA	Standard BCAA	
n	15	14	p
Age, median (IQR) years	82 (77–92)	80.5 (77.8–86.3)	ns
Female (Sex), n (%)	9 (60%)	9 (64.3%)	ns
GCS, median (IQR)	9 (7–14)	9.5 (6.25–13)	ns
BMI, median (IQR)	21.8 (17.6–23.5)	19.8 (16.4–22.9)	ns
**On admission**			
Alb, median (IQR)	3.5 (3–4.1)	3.3 (3–3.7)	ns
TTR, median (IQR)	12.7 (12–17.2)	10.7 (8.25–13.0)	ns
CRP, median (IQR)	1.02 (0.115–3.705)	0.673 (0.142–2.961)	ns
Cre, median (IQR)	0.78 (0.71–1.04)	0.695 (0.51–0.91)	ns
**On the 5th day**			
Alb, median (IQR)	2.6 (2.4–3.0)	2.6 (2.3–2.9)	ns
TTR, median (IQR)	11.8 (7.3–12.6)	9.65 (7.83–11.9)	ns
CRP, median (IQR)	5.243 (3.038–10.404)	4.077 (0.700–6.369)	ns
Cre, median (IQR)	0.74 (0.65–2.06)	0.63 (0.50–0.88)	< 0.05
**On the 7th day**			
Alb, median (IQR)	2.6 (2.4–2.9)	2.5 (2.2–2.8)	ns
TTR, median (IQR)	15.7 (10.2–20.2)	10.7 (8.3–12)	< 0.05
CRP, median (IQR)	4.774 (1.183–6.563)	2.459 (0.921–5.656)	ns
Cre, median (IQR)	0.79 (0.63–2.03)	0.62 (0.47–0.86)	ns
**Enteral nutrient (baseline)**			
Impact	1	3	
Peptamen AF	4	7	
Mein	7	3	
Renalen MP	3	1	

*BCAA* Branched chain amino acid, *GCS* Glasgow Coma Scale, *BMI* body mass index (kg/m^2^), *Cre* creatinine (mg/dl), *p* probability, *ns* not significant

**Table 3:** Patients charactreristics in leucine enriched and standard BCAA dietary supplements

Case	GCS on admission	Diagnosis	Medical history	BMI	BW	Cre on admission	eGFR on admission	T-CHO on admission	TG on admission	mRS before stroke	EN (1 pack)	Protein intake by EN (g/day)	Protein intake by EN (g/kg/day)	Calorie intake (/day)	Target calorie (/day)	Outcome	Hospitalization (days)
**Leucine enriched BCAA dietary supplement**																	
1	7	Cardioembolic stroke	Af, HT, Asthma	23.3	62	0.73	58.15	201	94	0	Impact (250 k, P14g)	70	1.1	1250	1515	Discharge	8
2	13	Ischemic stroke (Large vessel occlusion)	HT, DM, ASO, post-surgery of rectal cancer	24.5	65	0.78	74.03	241	203	0	Peptamen AF (300 k, P19g)	75.6	1.2	1200	1434	Discharge	15
3	15	Intracerebral hemorrhage (hypertensive)	HT, CRF (HD), hypothroidism	18.4	50	6.96	6.56	97	58	3	Renalen MP (400 k, P14g)	42	0.8	1200	1239	Discharge	11
4	9	Intracerebral hemorrhage (hypertensive)	HT, ASO	21.8	49	1.04	52.47	174	56	3	Peptamen AF (300 k, P19g)	75.6	1.5	1200	1122	Discharge	14
5	11	Cardioembolic stroke	HT, DM, CKD, Anemia	18.3	44	1.75	21.30	206	101	5	Renalen MP (400 k/P)	42	1.0	1200	1328	Discharge	12
6	12	Cardioembolic stroke	Af, HT, DM, MVR	17.6	45	0.74	57.51	139	42	0	Peptamen AF (300 k, P19g)	75.6	1.7	1200	1344	Discharge	8
7	7	Intracerebral hemorrhage (hypertensive)	HT, DL, CKD	13.9	30	0.71	56.44	286	65	3	MEIN (200 k, P10g)	60	2.0	1200	1005	Discharge	12
8	3	Intracerebral hemorrhage (hypertensive)	Paf, HT, DM, Dementia, DVT	23.5	64	0.68	80.80	159	87	2	MEIN (200 k, P10g)	70	1.1	1400	1803	In-hospital death	8
9	9	Ischemic stroke (Large vessel occlusion)	HT, DL, CKD, CHF	20.6	44	0.86	45.76	256	124	5	MEIN (200 k, P10g)	60	1.4	1200	1081	Discharge	14
10	5	Intracerebral hemorrhage (hypertensive)	HT, DL, ASO, OMI (post-CABG), post-CAS	23.4	58	0.92	50.61	137	167	0	MEIN (200 k, P10g)	70	1.2	1400	1356	Discharge	10
11	14	Ischemic stroke (Large vessel occlusion)	Af, HT, DM, DL, CRF, post-CABG, stent gradt for AAA	22.3	57	9.35	4.75	119	92	3	MEIN (200 k, P10g)	70	1.2	1400	1521	Discharge	17
12	15	Cardioembolic stroke	Af, HT, left IC occlusion, breast cancer	25.7	57	0.59	71.84	216	65	0	MEIN (200 k, P10g)	70	1.2	1400	1563	Discharge	13
13	7	Cardioembolic stroke	Af, HT, DL, IGT, Lung cancer, DVT, intracerebral aneurysm (unruptured)	23.5	55	0.76	56.39	158	102	0	Renalen MP (400 k, P14g)	42	0.8	1200	1293	In-hospital death	21
14	14	Cardioembolic stroke	Paf	15.8	37	0.70	59.18	188	128	3	Peptamen AF (300 k, P19g)	75.6	2.0	1200	1063	Discharge	16
15	8	Cardioembolic stroke	HT, right MCA stenosis	17.3	39	0.83	47.72	210	58	4	MEIN (200 k, P10g)	50	1.3	1000	943	Discharge	8
Median	9			21.8	50	0.78	56.4	188	92	3		70	1.2	1200	1328		12
**Standard BCAA dietary supplement**																	
1	7	Ischemic stroke (Large vessel occlusion)	HT, DL, Paf	22.6	67	0.77	71.7	206	36	0	Impact (250 k, P14g)	70	1.0	1500	1628	Discharge	10
2	13	Cardioembolic stroke	Af, HT, DM, Obesity, OCI	25.8	62	0.49	92.8	161	63	0	Peptamen AF (300 k, P19g)	75.6	1.2	1200	1321	Discharge	11
3	14	Cardioembolic stroke	DM, OCI, AAA	16.5	45	0.65	91.1	159	105	2	Peptamen AF (300 k, P19g)	94.5	2.1	1500	1350	Discharge	14
4	3	Cardioembolic stroke	HT, DM, HL, CABG,OCI, Basedow,	19.5	44	1.15	35.2	133	90	4	MEIN (200 k, P10g)	60	1.4	1200	1077	Discharge	8
5	4	Cardioembolic stroke	Paf, HT, HL, DM	23.8	50	0.9	44.4	235	116	2	Impact (250 k, P14g)	56	1.1	1000	1036	Discharge	9
6	13	Ischemic stroke (small vessel occlusion)	HT, AP, OCI, Dementia, DVT	22.0	55	0.68	60.9	241	85	4	MEIN (200 k, P10g)	60	1.1	1200	1286	Discharge	10
7	12	Intracerebral hemorrhage (hypertensive)	HT, DVT	20.0	45	0.71	58.1	205	133	3	MEIN (200 k, P10g)	50	1.1	1000	1258	Discharge	10
8	8	Cardioembolic stroke	Af, HT, OCI, DL	24.0	54	0.47	92.4	206	113	4	Peptamen AF (300 k, P19g)	75.6	1.4	1200	1340	Discharge	9
9	11	Intracerebral hemorrhage (hypertensive)	HT	14.5	39	0.48	91.7	182	34	0	Impact (250 k, P14g)	56	1.4	1000	1054	Discharge	9
10	8	Ischemic stroke (Large vessel occlusion)	HT, DM, DL	21.8	63	0.6	97.5	223	98	4	Peptamen AF (300 k, P19g)	94.5	1.5	1500	1590	Discharge	18
11	3	Cardioembolic stroke	Paf, HT,LC	15.9	43	1.68	30.8	126		1	Renalen MP (400 k, P14g)	35	0.8	1000	1111	In-hospital death	10
12	9	Ischemic stroke (Large vessel occlusion)	HT,DM,OCI, DL, Dydrocephalus, ASO	18.7	42	0.72	56.8	189	171	5	Peptamen AF (300 k, P19g)	75.6	1.8	1200	1238	Discharge	7
13	10	Ischemic stroke (small vessel occlusion)	HT, SAH	15.0	36	0.52	83.7	163	96	4	Peptamen AF (300 k, P19g)	75.6	2.1	1200	1099	Discharge	7
14	13	Cardioembolic stroke	HT, HL, DM	18.1	48	0.92	60.6	159	75	0	Peptamen AF (300 k, P19g)	75.6	1.6	1200	1218	Discharge	22
Median	9.5			19.7	46.5	0.70	66.3	186	96	3		72.8	1.4	1200	1248		10

*BCAA* branced-chain amino acids, *P* protein, *GCS* Glasgow Coma Scale, *BMI* body mass index (kg/m2), *BW* body weight (kg), *Cre* serum creatinine (mg/dl), *eGFR* estimated Glomerular Filtration Rate (mL/min/1.73 m2), *T-CHO* total cholesterol (mg/dl), *TG* triglyceride (mg/dl), *mRS* modified Rankin scale, *EN* enteral nutrient, *k* kcal, *Af* atrial filtration, *HT* hypertension, *DM* diabetes mellitus, *CRF* chronic renal failure, *HD* hemodyalysis, *ASO* arteriosclerosis obliterans, *CKD* chronic kidney disease, *MVR* mitral valve replacement, *DL* dyslipidemia, *DVT* deep venous thrombosis, *CHF* chronic heart failure, *OMI* old myocardial infarction, *Paf* paroxysmal af, *CABG* coronary aorta bypass surgery, *CAS* carotid artery stenting, *AAA* abdominal aorta aneurysm, *IC* internal carotid artery, *IGT* impaired glucose tolerance, *MCA* middle cerebral artery

**Table 4:** Changes of serum markers in leucine enriched and standard BCAA dietary supplements

Case	TTR on admission	TTR on the fifth day	TTR on the seventh day	Alb on admission	Alb on the fifth day	Alb on the seventh day	CRP on admission	CRP on the fifth day	CRP on the seventh day	Cre on admission	Cre on the fifth day	Cre on the seventh day
**Leucine enriched BCAA dietary supplement**												
1	13.1	14.0	23.1	4.0	2.9	3.2	8.831	3.038	1.183	0.73	0.53	0.52
2	16.1	10.8	15.7	4.3	3.1	2.6	0.870	16.376	6.161	0.78	1.25	1.16
3	12.0	7.3	11.3	3.0	2.3	2.5	1.862	10.404	4.774	6.96	5.67	5.79
4	12.3	3.5	4.4	4.1	2.2	2.0	0.324	38.079	21.280	1.04	3.97	2.03
5	12.7	12.4	13.3	2.8	2.1	2.1	1.020	2.160	0.941	1.75	2.06	1.83
6	9.0	7.3	10.2	3.0	2.6	2.4	3.705	5.431	2.131	0.74	0.74	0.79
7	17.2	12.4	20.5	3.5	2.6	2.7	0.036	2.009	0.385	0.71	0.69	0.62
8	7.9	6.7	8.2	3.4	2.5	2.6	8.168	24.719	15.136	0.68	0.92	2.09
9	8.2	8.4	17.2	3.2	2.4	2.6	1.673	8.285	1.457	0.86	0.70	0.65
10	24.7	11.9	10.9	4.2	2.7	2.1	0.016	6.469	5.849	0.92	1.01	0.82
11	16.9	13.3	20.2	4.1	3.1	3.0	0.273	5.243	4.605	9.35	4.74	5.34
12	23.3	12.7	19.6	4.2	2.8	2.9	0.093	4.078	1.045	0.59	0.54	0.63
13	12.5	12.6	20.4	3.3	3.0	3.4	9.265	5.199	8.887	0.76	0.56	0.64
14	17.8	11.8	15.9	2.9	2.4	2.5	1.212	4.066	6.563	0.70	0.65	0.50
15	12.2	10.7	8.1	3.7	3.4	2.9	0.115	0.598	6.046	0.83	0.70	0.64
Median	12.9	11.9	15.8	3.5	2.6	2.6	1.116	5.337	4.690	0.77	0.83	0.81
MDB A-5		−3.6 (*p* < 0.001)			−0.9 (*p* < 0.0001)			4.0 (*p* < 0.01)			−0.05 (ns)	
MDB 5–7			4.1 (*p* < 0.001)			0.1 (ns)			−1.9 (*p* < 0.05)			−0.05 (ns)
**Standard BCAA dietary supplement**												
1	11.8	11.0	10.4	4.0	2.6	2.4	1.332	5.301	3.268	0.77	0.72	0.70
2	8.1	10.4	11.1	2.9	2.3	2.2	3.796	0.555	0.791	0.49	0.54	0.65
3	8.3	8.3	12.1	3.0	2.5	2.7	0.822	5.924	1.721	0.65	0.51	0.51
4	8.0	9.5	10.1	3.0	2.0	2.1	3.388	4.058	2.419	1.15	1.10	1.08
5	10.4	8.4	8.9	3.7	2.6	2.7	0.407	3.396	2.499	0.9	0.85	0.87
6	20.3	13.2	13.6	3.7	3.3	3.1	0.050	0.149	0.210	0.68	0.63	0.64
7	17.1	13.6	12.0	3.5	2.9	2.7	0.028	4.327	5.864	0.71	0.62	0.60
8	5.8	6.2	6.6	2.8	2.3	2.3	8.327	4.095	5.587	0.47	0.41	0.42
9	12.8	12.8	12.0	4.4	3.5	3.4	0.524	0.748	0.984	0.48	0.45	0.40
10	10.6	9.8	10.9	2.9	2.3	2.2	2.345	7.703	3.041	0.60	0.40	0.39
11	10.7	3.1	4.1	3.2	1.5	1.5	0.172	23.428	18.559	1.68	1.61	1.85
12	12.4	11.6	12.0	3.4	3.0	2.9	0.227	0.227	0.964	0.72	0.64	0.57
13	8.8	8.8	10.5	3.0	2.6	2.6	0.015	0.812	0.196	0.52	0.52	0.48
14	13.5	6.4	4.1	3.5	2.5	2.2	2.819	30.905	33.414	0.92	0.97	0.85
Median	10.7	9.7	10.7	3.3	2.6	2.5	0.673	4.077	2.459	0.70	0.63	0.62
MDB A-5		−0.8 (< 0.05)			−0.6 (*p* < 0.0001)			1.893 (*p* < 0.05)			−0.015 (*p* < 0.01)	
MDB 5–7			0.45 (ns)			−0.1 (ns)			−0.278 (ns)			− 0.05 (ns)

*BCAA* branched-chain amino acids, *TTR* transthretin (mg/dl), *Alb* albumin (g/dl), *CRP* high-sensitivity C-reactive protein (mg/dl), *Cre* creatinine (mg/dl), *MDB A-5* median difference between on admission and the fifth day, *MDB 5–7* median difference between on the fifth and the seventh day, *p* probability, *ns* not significant

In LEBDs group, the median creatinine (Cre) and estimated glomerular filtration rate (eGFR) on admission were 0.78 (mg/dl), and 56.4 (mL/min/1.73m^2^), respectively. In 12 of the 15 patients, eGFR was less than 60 (mL/min/1.73 m2). The 12 patients suffered from renal dysfunction on admission (Table 3). In LEBDs group, the median target calorie and real calorie intake by EN was 1328 and 1200 kcal/day, respectively. The calorie status was 90.4% of the plan on the fifth day. We used protein-rich EN except for REANALEN MP in 12 of the 15 patients (Table 3). In SBDs group, their median Cre and eGFR, on admission were 0.70 (mg/dl), and 66.3 (mL/min/1.73 m2). In 5 of the 14 patients, eGFR was less than 60 (mL/min/1.73m^2^). The 5 patients suffered from renal dysfunction on admission (Table 3). In SBDs group, the median target calorie and real calorie intake by EN was 1248 and 1200 kcal/day, respectively. The calorie status was 96.2% of the plan on the fifth day. We used protein-rich EN except for REANALEN MP in 13 of the 14 patients (Table 3).

Overall renal function did not deteriorate except for two cases in LEBDs group (Table 4). In Case 2 and 4 in LEBDs group, serum Cre level acutely rose on the fifth day, and protein per day by PEPTAMEN AF was reduced from 75.6 to 56.7 g from the fifth day. The LEBDs was added on protein of 56.7 g by PEPTAMEN AF in case 2 and 4, and their Cre level did not deteriorate on the seventh day (Tables 3 and 4).

In LEBDs, 13 (86.7%) of the 15 patients discharged within 17 days, and they were transferred to comprehensive rehabilitation centers after their TTR and CRP were improving. Two of the 15 patients died on the eighth day and the twenty-first day, respectively. Median hospitalization in LEBDs was 12 days. In SBDs, 12 (85.7%) of the 14 patients discharged within 17 days, and they were transferred to comprehensive rehabilitation centers, although neither TTR nor CRP were not improving. One of the 14 patients died on the tenth day. Median hospitalization in SBDs was 10 days (Table 3).

## Discussion

Our results demonstrate that quick improvements on the transthyretin and CRP level were observed in acute stroke patients who received leucine enriched BCAA dietary supplement added to baseline enteral nutrients.

Our patients were very elderly, suffered from impaired consciousness and renal dysfunction, and malnutrition within the 5 days of admission. We used EN containing leucine and MCT and provided protein of median 1.2 g or more (/kg/day) in the 29 patients [11]. Their TTR and Alb level, however, declined. Serum Cre acutely rose in two of the 25 patients administered with protein-rich EN, and protein-rich EN-induced deterioration of renal function, therefore, must be considered. We administered the LEBDs in the 15 patients, and 13 of them achieved an early discharge to comprehensive rehabilitation centers within 17 days after TTR and CRP were improving. Their clinical course was not usual. Thirteen of the 14 patients with SBDs survived and were transferred to comprehensive rehabilitation hospitals without improvements of TTR and CRP. SBDs might contribute to prevention of further deterioration of TTR, Alb and CRP, although SBDs could not improve them.

We supposed that the LEBDs had a quick effect on improvement of hypoproteinemia and cessation of inflammation in the 15 patients. After administration of the LEBDs, the TTR level rose soon, and the CRP level decreased soon, probably because leucine enriched BCAA played critical roles in the regulation of energy homeostasis, nutrition metabolism, gut health, immunity as they acted as potential anti-inflammatory mediators [26–28], further helped attenuate inflammation and enhance protein synthesis [13, 27, 28]. The Alb level did not change soon after the administration of the LEBDs, as the Alb level usually improves slowly. The LEBDs likely provides benefits with malnutrition patients in a stroke care unit or intensive care unit.

### Study limitations

Our study had several limitations. Our study included a small number of patients. Our study was not a prospective randomized controlled study but a retrospective observational cohort study of routine medical care. We, therefore, did not have a placebo-controlled group without BCAA supplement, and we compared LEBDs group with SBDs group. The LEBDs contained MCT or n-3 fatty acids; no information, however, was provided in SBDs. Our patients started to use different types of enteral nutrients as the baseline. All patients were likely of Japanese ancestry; therefore, not representative of the general population. Our study investigated short-term outcomes of blood examinations, in-hospital death, and hospitalization days but did not study the long-term effect. To confirm our results, therefore, future prospective studies are required in patients with the standardization of enteral nutrients.

## Conclusion

In acute stroke patients receiving leucine enriched BCAA dietary supplement, quick improvements in transthyretin and CRP were observed compared to patients with standard BCAA dietary supplement. The results warrant the further clinical application of the LEBDs in acute patients with malnutrition.

## Data Availability

The datasets analyzed during the current study are available from the corresponding author on reasonable request.

## Abbreviations

Alb: Albumin
BCAA: Branched-chain amino acid
BMI: Body mass index
BW: Body weight
Cre: Creatinine
CRP: C-reactive protein
eGFR: estimated glomerular filtration rate
EN: Enteral nutrient
ETF: Enteral tube feeding
LEBDs: Leucine enriched BCAA dietary supplement
SBDs: Standard BCAA dietary supplement
TTR: Transthyretin
