# Correction to: Quick and effective improvement of leucine enriched dietary supplement on malnutrition in acute stroke patients receiving enteral tube feeding

**DOI:** 10.1186/s12873-020-00393-0

**Published:** 2020-12-29

**Authors:** Takahisa Mori, Kazuhiro Yoshioka

**Affiliations:** Department of Stroke Treatment, Shonan Kamakura General Hospital Stroke Center, Okamoto 1370-1, Kamakura City, Kanagawa 247-8533 Japan

**Correction to: BMC Emerg Med 20, 56 (2020)**

**https://doi.org/10.1186/s12873-020-00351-w**

The Tokushukai Group Ethical Committee approved that our study had added patients with standard BCAA for a control group to patients with leucine enriched BCAA and additional opt-out consent of patients in the control group is obtained (TGE01445-024).
